# Emergency department providers’ perspectives on patients’ use of cannabis: therapeutic potential, risks, and prescribing experience

**DOI:** 10.1186/s12873-026-01710-9

**Published:** 2026-08-07

**Authors:** Lisa Moore, Justin Gawaziuk, Shaan Sareen, Brenda Comaskey, Rae Spiwak, Sarvesh Logsetty

**Affiliations:** 1 Department of Surgery, Max Rady Faculty of Health Sciences, University of Manitoba, Winnipeg, Canada; 2 Department of Psychiatry, Max Rady College of Medicine, University of Manitoba, Winnipeg, MB Canada; 3 Department of Pediatrics and Child Health, Max Rady College of Medicine, University of Manitoba, Winnipeg, MB Canada; 4 Manitoba Centre for Health Policy, Max Rady College of Medicine, University of Manitoba, Winnipeg, MB Canada

**Keywords:** Medical cannabis, Emergency medicine, Provider attitudes, Cannabis prescribing, Survey research

## Abstract

**Background:**

Cannabis legalization in Canada has increased both therapeutic and recreational use. Emergency department (ED) providers encounter patients using cannabis recreationally, to self-manage symptoms, or under medical authorization, often without a clear distinction between these contexts at the point of care. However, provider attitudes toward cannabis in the emergency setting remain poorly understood, and no Canadian studies have specifically explored this group. This knowledge gap limits the development of clinical guidance and education for ED staff.

**Methods:**

We conducted a cross-sectional survey of physicians and nurses working in the Adult Emergency Department at the Health Sciences Centre (HSC), Winnipeg, Manitoba, from October 2024 to February 2025. The survey evaluated attitudes toward legalization and medical use, perceived legal, social, and health risks, and prescribing experience. Descriptive statistics were used. Survey reporting followed best practice guidelines.

**Results:**

Forty-five providers completed the survey. Most (71.1%) supported legalization, and 86.7% believed cannabis has therapeutic value. Nearly all (95.6%) agreed that eligible patients should have access to cannabis. Legal (91.1%) and social (77.3%) risks were perceived as low, while views on health risks varied; 38.6% perceived moderate or high health risks. No participants had prescribed cannabis, although 42.2% were in prescribing roles.

**Conclusion:**

This is the first study to examine Canadian ED provider attitudes toward cannabis. While most respondents supported legalization (71.1%) and endorsed the therapeutic potential of cannabis (86.7%), with nearly all (95.6%) agreeing that eligible patients should have access, prescribing experience was limited, and many of those surveyed held uncertain or mixed views on cannabis safety, particularly relative to prescription pain medications. These findings highlight the need for targeted education, clinical tools, and evidence-based guidance to support cannabis-related care in emergency settings.

**Supplementary Information:**

The online version contains supplementary material available at https://doi.org/10.1186/s12873-026-01710-9.

## Introduction

Since 2001, medical cannabis has been legally available in Canada under a restricted access framework [1]. In 2018, the federal government passed the *Cannabis Act* (Bill C-45), legalizing recreational cannabis for adults [2]. This legislative shift increased access and product diversity, contributing to rising use among Canadian adults, with the most striking relative increase in people aged 65 and older [3, 4]. By 2020, one in five Canadians reported cannabis use, a substantial increase from pre-legalization levels [4]. In 2023, 10% of Canadians reported using cannabis to manage medical issues such as chronic pain, anxiety, and sleep disturbances; however, only 18% reported having a medical document from a healthcare provider, suggesting a growing reliance on self-directed medical use [5].

Ongoing stigma, provider knowledge gaps, and clinician discomfort discussing cannabis may lead many Canadians to seek information outside the healthcare system [6, 7]. For example, older adults often turn to friends, the internet, or cannabis store employees for guidance on use [6]. In the context of a worsening opioid crisis in Canada, cannabis is increasingly explored as a potential non-opioid alternative for pain management, further emphasizing the need for provider education and clinical guidance on its therapeutic use [8, 9].

Provider attitudes and knowledge influence how medical cannabis is incorporated into clinical practice [10–15]. Understanding the perspectives of emergency department (ED) clinicians is particularly important, as they occupy a unique role. ED staff are encountering more frequent presentations related to the adverse effects of cannabis use (such as cannabinoid hyperemesis syndrome (CHS), psychosis, and accidental ingestion); these patients may be consuming cannabis recreationally, to self-manage symptoms, or using it under medical authorization, and the context of their use may not be clear to clinicians [16–20]. Emergency providers also treat patients with conditions that may benefit from cannabis such as chronic pain [5] or chemotherapy-related nausea [20]. However, no published Canadian studies have examined the attitudes of emergency medicine clinicians toward cannabis. This study aims to explore provider attitudes, risk perceptions, and prescribing experience related to cannabis in a Canadian tertiary care ED, to help guide the development of targeted education and support evidence-informed cannabis-related care in emergency settings.

## Methods

### Study design and time period

We conducted a cross-sectional survey between October 2024 and February 2025.

### Study setting

This study took place in the Adult Emergency Department at the Health Sciences Centre (HSC) in Winnipeg, Manitoba. This facility serves patients aged 17 years and older.

### Population

Eligible participants included physicians and nurses working in the facility’s Adult Emergency Department. Invitations were distributed to all physicians and nurses on the department’s internal email list. Participation was voluntary and anonymous. No identifying information was collected, and responses were stored securely within a Microsoft Forms document hosted on the institution’s cloud-based email platform. We were unable to determine a precise response rate, as the total number of ED staff who received the survey invitation was unknown. However, based on an estimated denominator of 154 staff provided by ED site leadership (48 emergency room physicians, 31 residents, and roughly 75 nurses), the response rate was approximately 29%, which is consistent with other surveys of health care workers using email as a mode of recruitment [21].

### Questionnaire development

The survey was adapted from an instrument developed by Stillman et al. [22], originally designed to assess cannabis-related knowledge and attitudes among individuals with spinal cord injury. Our group initially adapted this instrument for use with a Canadian burn survivor surgical population. We selected this instrument because it includes items about cannabis-related attitudes and beliefs in a surgical setting. For use in the present study, we adapted that instrument, retaining items relevant to attitudes and beliefs about cannabis. Other instruments have been used to survey medical providers about cannabis; Philpot et al. surveyed primary care providers’ attitudes, beliefs, and knowledge about medical cannabis within a state-regulated cannabis program in the US [12], and Kruger et al. examined physicians’ practice patterns, self-rated competence, and attitudes toward medical cannabis in a US health system setting [23]. In contrast, our survey also assessed general attitudes toward cannabis without restricting items to a medical authorization context. Stillman’s survey was informed by prior studies, including validated tools such as the DFAQ-CU (Daily Sessions, Frequency, Age of Onset, and Quantity of Cannabis Use Inventory), although no formal validation of the instrument was reported. Our brief, anonymous questionnaire (estimated completion time: 2–3 min) was distributed via an internal department email link.

### Outcome measures

The survey evaluated attitudes toward legalization, therapeutic potential, and patient access to cannabis. It also assessed perceived risks to cannabis users (legal, social, and health-related) and prescribing experience.

### Data analysis

Survey data was collected in Microsoft Forms. We used descriptive statistics to summarize responses. Likert-scale and risk perception items were reported as percentages. Prescriber status and cannabis prescribing experience were reported categorically. Differences in item responses between prescribers and non-prescribers were assessed using the Fisher-Freeman-Halton exact test for three-category items and Fisher’s exact test for dichotomous items; two-sided p-values are reported, with significance set at *p* < 0.05. Analyses were conducted in R (version 4.6.0; R Core Team, Vienna, Austria).

### Sample size

A total of 45 ED providers completed the survey. No formal sample size calculation was performed.

### Reporting guidelines

This study followed Kelley et al.’s best practice guidelines for survey research, including clear objectives, defined population, and ethical approval [24]. Although the survey drew on components of previously validated instruments, the adapted tool has not undergone formal psychometric validation, and no pilot testing was performed in this setting.

### Ethics approval

The study was reviewed and approved by the University of Manitoba’s Bannatyne Campus Research Ethics Board (H2021-197). Informed consent was obtained from all participants prior to survey completion.

## Results

### Participant characteristics

A total of 45 ED providers completed the survey. Of these, 19 (42.2%) were prescribers (e.g., physicians or nurse practitioners) and 25 (55.6%) were non-prescribers; one participant (2.2%) did not indicate prescriber status (Table 1). That participant completed all survey items and is included in overall responses but excluded from the subgroup comparisons. No prescriber reported having prescribed cannabis.

**Table 1:** ED Provider Prescriber Status (*N* = 45)

Response	*n* (%)
Prescriber	19 (42.2)
Non-prescriber	25 (55.6)
No response	1 (2.2)

Prescriber denotes a provider with legal authority to independently prescribe medications (general prescribing authority, not specific to cannabis). Survey item: “Have you prescribed cannabis for medical use by patients?” (response options: Yes / No / I am not a prescriber). “Yes” and “No” denote prescribers generally; “I am not a prescriber” denotes non-prescribers. No person with prescribing privileges had specifically prescribed cannabis (0/19)

### Attitudes toward cannabis

71.1% of participants supported cannabis legalization, and 86.7% believed cannabis has therapeutic potential (Table 2). Nearly all respondents (95.6%) agreed that patients who may benefit should have access to medical cannabis. Few participants viewed cannabis as dangerous: 11.1% considered it a gateway drug, 4.4% believed it carried a high overdose risk, and 2.2% described it as very dangerous. However, opinions were mixed regarding the relative safety of prescription painkillers compared to cannabis: 11.1% agreed that prescription analgesics are safer, 48.9% were neutral, and 40.0% disagreed.

**Table 2:** ED Provider Attitudes Toward Cannabis Use (*N* = 45)

Statement	Agree *n* (%)	Neutral *n* (%)	Disagree *n* (%)
Cannabis should be legalized	32 (71.1)	10 (22.2)	3 (6.7)
Cannabis smokers are prone to violence	6 (13.3)	10 (22.2)	29 (64.4)
Withdrawal from cannabis can be life-threatening	2 (4.4)	10 (22.2)	33 (73.3)
Cannabis is a gateway drug	5 (11.1)	13 (28.9)	27 (60.0)
Cannabis use has a high risk of overdose	2 (4.4)	7 (15.6)	36 (80.0)
Cannabis is a very dangerous drug	1 (2.2)	5 (11.1)	39 (86.7)
Cannabis could have medicinal effects	39 (86.7)	4 (8.9)	2 (4.4)
Prescription painkillers are safer	5 (11.1)	22 (48.9)	18 (40.0)
Medical cannabis should be available	43 (95.6)	2 (4.4)	0 (0.0)

### Perceptions of risk

Most participants perceived the legal (91.1%) and social (77.3%) risks of cannabis use to be mild or non-existent (Table 3). Opinions about potential health risks were more divided: 61.4% reported mild or no risk, while 38.6% believed there to be moderate or great risk to the health of cannabis users.

**Table 3:** ED Provider Perceived Risks of Cannabis Use (*N* = 45)

Risk Type	No/Mild *n* (%)	Moderate/Great *n* (%)
Legal Risk	41 (91.1)	4 (8.9)
Social Risk	34 (77.3)	10 (22.7)
Health Risk	27 (61.4)	17 (38.6)

^a^One participant did not respond to social and health risk items (*n* = 44)

### Attitudes by prescriber status

For the purposes of this study, a prescriber was defined as a provider with legal authority to independently prescribe medications; all other emergency staff were classified as non-prescribers. This role-based status is distinct from cannabis-prescribing behaviour: no respondent (0/45) had prescribed cannabis. Prescriber status was determined from the survey item “Have you prescribed cannabis for medical use by patients?“, with response options “Yes,” “No,” and “I am not a prescriber.”

Support for legalization was high among both prescribers and non-prescribers (73.7% vs. 72.0%). Both groups also saw cannabis as a potential therapeutic option (84.2% vs. 88.0%) and approved of access to cannabinoids for patients (89.5% vs. 100.0%) (Table 4). We found considerable uncertainty among prescribers about the safety of cannabis versus prescription painkillers, with 63.2% reporting a neutral stance (vs. 40.0% of non-prescribers). None of the prescribers (0.0%) believed opioids were safer than cannabis, compared to 16.0% of non-prescribers. This ambivalence suggests a tension between prescribers’ awareness of the risks of opioids and the lack of clear guidance about the practical applications of cannabis for pain.

**Table 4:** Attitudes Toward Cannabis Use by Prescriber Status (*n* = 44)

Statement	Response	Prescribers (*n* = 19) n (%)	Non-Prescribers (*n* = 25) n (%)	*p*
Cannabis should be legalized	Agree	14 (73.7)	18 (72.0)	0.586
	Neutral	3 (15.8)	6 (24.0)	
	Disagree	2 (10.5)	1 (4.0)	
Cannabis smokers prone to violence	Agree	2 (10.5)	3 (12.0)	0.424
	Neutral	6 (31.6)	4 (16.0)	
	Disagree	11 (57.9)	18 (72.0)	
Withdrawal can be life threatening	Agree	0 (0.0)	2 (8.0)	0.299
	Neutral	3 (15.8)	7 (28.0)	
	Disagree	16 (84.2)	16 (64.0)	
Cannabis is a gateway drug	Agree	0 (0.0)	4 (16.0)	0.151
	Neutral	5 (26.3)	8 (32.0)	
	Disagree	14 (73.7)	13 (52.0)	
Cannabis has a high risk of overdose	Agree	0 (0.0)	2 (8.0)	0.087
	Neutral	1 (5.3)	6 (24.0)	
	Disagree	18 (94.7)	17 (68.0)	
Cannabis is a very dangerous drug	Agree	0 (0.0)	1 (4.0)	0.796
	Neutral	3 (15.8)	2 (8.0)	
	Disagree	16 (84.2)	22 (88.0)	
Cannabis has medicinal potential	Agree	16 (84.2)	22 (88.0)	0.275
	Neutral	1 (5.3)	3 (12.0)	
	Disagree	2 (10.5)	0 (0.0)	
Prescription painkillers are safer	Agree	0 (0.0)	4 (16.0)	0.121
	Neutral	12 (63.2)	10 (40.0)	
	Disagree	7 (36.8)	11 (44.0)	
Medical cannabis should be available	Agree	17 (89.5)	25 (100.0)	0.181
	Neutral	2 (10.5)	0 (0.0)	
	Disagree	0 (0.0)	0 (0.0)	

^a^p-values from the Fisher-Freeman-Halton exact test. One participant did not indicate prescriber status (*n* = 44). Responses from this individual are included in overall totals (Tables 1, 2 and 3) but excluded from stratified results (Tables 4 and 5); therefore, there are minor discrepancies between overall and subgroup counts

Prescribers perceived lower legal risks (100.0% vs. 88.0%) and social risks (83.3% vs. 76.0%) associated with cannabis use versus non-prescribers (Table 5). Both groups had similar perceptions of health risks, with approximately two-thirds of each rating health risks as non-existent or mild. One prescriber did not respond to the social and health risk items.

**Table 5:** Perceived Risk of Cannabis Use by Prescriber Status (*n* = 44)

Risk Category	Group	No/Mild *n* (%)	Moderate/Great *n* (%)	*p*
Legal Risk	Prescribers	19 (100.0)	0 (0.0)	0.247
	Non-Prescribers	22 (88.0)	3 (12.0)	
Social Risk	Prescribers	15 (83.3)	3 (16.7)	0.712
	Non-Prescribers	19 (76.0)	6 (24.0)	
Health Risk	Prescribers	12 (66.7)	6 (33.3)	0.755
	Non-Prescribers	15 (60.0)	10 (40.0)	

^a^ p-values from Fisher’s exact test. One prescriber did not respond to the social and health risk items (*n* = 18 for those rows). Responses from the participant who did not indicate prescriber status are included in overall totals (Tables 1, 2 and 3) but excluded from stratified results (Tables 4 and 5); therefore, there are minor discrepancies between overall and subgroup counts

## Discussion

### Interpretation of findings

ED providers in our sample expressed broad support for both cannabis legalization and its therapeutic use. Most respondents believed patients should have access to medical cannabis, yet none reported having prescribed it, and many were uncertain about its safety compared to prescription pain medications, particularly prescribers. These responses reveal a disconnect between optimism toward cannabis and limited practical clinical preparedness in the ED context.

### Comparison to previous studies

Our findings are consistent with previous Canadian and U.S. studies, which report generally favorable attitudes toward cannabis use among physicians, pharmacists, nurses, and medical trainees, while also highlighting persistent knowledge gaps and limited clinical confidence [10–15]. However, emergency providers remain underrepresented in the existing literature.

### Strengths and limitations

A key strength of this study is its novel focus on a population that has been thus far excluded from Canadian cannabis research. By including both physicians and nurses, the study provides a broader perspective on cannabis-related attitudes within emergency care. Our setting, a major urban tertiary care hospital, enhances the clinical relevance of the findings by reflecting the complexity and diversity of cases seen in acute care.

This study has several limitations. Our survey was completed by a small number of participants (*n* = 45) from a single emergency department, which may limit the generalizability of the findings. Our questionnaire was adapted from a tool that had not undergone formal validation and was not pilot-tested or psychometrically assessed in the emergency room setting. Providers who are less familiar or comfortable with the topic of cannabis may be less likely to participate, potentially introducing selection bias toward more favorable views. As a tertiary centre, this department regularly cares for patients whose cannabis use is recreational or otherwise outside a medical authorization, and clinicians’ views here may reflect that broader exposure. Our survey instrument did not capture provider perspectives on condition-specific harms frequently seen in emergency care, such as CHS, cannabis-associated psychosis, or other potential psychiatric outcomes such as anxiety. Provider attitudes toward therapeutic cannabis may differ substantially from their views on cannabis use contributing to these acute presentations. The response rate was approximate, as the denominator was estimated and the nurse staffing list may have been out of date.

### Clinical implications

ED clinicians are encountering more patients who use cannabis for therapeutic purposes, yet many remain uncertain about its clinical applications. Clear, evidence-based guidance is needed to help providers navigate cannabis-related conversations, assess therapeutic potential, and address patient questions with confidence. Integrating cannabis into acute care practice may support symptom relief in areas such as pain and nausea, and in some cases, contribute to reducing reliance on opioids [8]. To ensure safe and consistent use, cannabis-specific clinical guidelines should be developed for emergency settings, complemented by targeted education that addresses pharmacology, potential drug interactions, risk mitigation, and prescribing considerations.

Qualitative research in emergency settings indicates patients presenting with cyclic vomiting and chronic cannabis use may not recognize the relationship between their cannabis consumption and CHS symptoms, attributing episodes instead to diet, alcohol intake, stress, or underlying gastrointestinal issues [25]; this uncertainty suggests the emergency department is an opportune context for providers to make the link between use and symptoms and provide education about CHS.

### Research implications

As one of the first Canadian studies to examine ED provider perspectives on cannabis, our survey opens the door for broader, multi-centre investigations that can further inform emergency medicine education, policy, and clinical practice. Future research should evaluate cannabis-focused training for emergency providers. Qualitative interviews with emergency department staff could identify and describe perceived risks of cannabis use and explore how cannabis-related harms are recognized and managed in the emergency setting. Future research should estimate how often emergency presentations involve self-directed versus medically authorized cannabis use, and how the clinical needs of these groups differ, to help develop practice guidelines. Medical education curricula should also be evaluated for cannabis content, as prior research has identified significant deficits in cannabis-related education and training for Canadian physicians [15]. Expanding research in medical cannabis may also drive the development of safe, standardized, and titratable formulations, such as metered-dose inhalers or precisely dosed oral products, specifically designed to meet the clinical demands of acute care environments.

## Conclusion

This study is the first to examine attitudes toward cannabis among Canadian emergency department providers. While participants expressed support for legalization (71.1%) and therapeutic use (86.7%), none had prescribing experience, and many held uncertain or mixed views on cannabis safety, particularly in comparison to prescription pain medications. These findings highlight the need for targeted cannabis education and the development of clear, evidence-based clinical guidelines. Addressing these gaps may enhance provider confidence, reduce reliance on opioids, and improve the quality of cannabis-related care in emergency settings.

## Supplementary Information

Below is the link to the electronic supplementary material.

Supplementary Material 1

## Data Availability

The datasets related to this study are available from the corresponding author on reasonable request.

## Abbreviations

ED: Emergency department
HSC: Health Sciences Centre
CHS: Cannabinoid hyperemesis syndrome

## References

[1] Government of Canada. Marihuana Medical Access Regulations (SOR/2001 – 227). Ottawa: Justice Laws Website; 2001 [Cited 2026 Jun 17]. Available from: https://laws-lois.justice.gc.ca/eng/regulations/sor-2001-227/.

[2] Government of Canada. Cannabis, Act (S.C. 2018, c. 16). Ottawa: Justice Laws Website; 2018 [Cited 2025 Aug 26]. Available from: https://laws-lois.justice.gc.ca/eng/acts/C-24.5/.

[3] Rotermann M. What has changed since cannabis was legalized? Health Rep. 2020;31(2):11–20. 10.25318/82-003-x202000200002-eng. 32073644

[4] Statistics Canada. Looking back from 2020: how cannabis use and related behaviours changed in Canada. Health Rep. 2021;32(4):3–14. 10.25318/82-003-x202100400001-eng. 33881274

[5] Health Canada. Canadian Cannabis Survey 2023: Summary. Ottawa: Government of Canada; 2023 [Cited 2025 Aug 26]. Available from: https://www.canada.ca/en/health-canada/services/drugs-medication/cannabis/research-data/canadian-cannabis-survey-2023-summary.html

[6] Butler JI, Dahlke S, Devkota R, et al. The information-seeking behavior and unmet knowledge needs of older medicinal cannabis consumers in Canada: a qualitative descriptive study. Drugs Aging. 2023;40(5):427–38. 10.1007/s40266-023-01030-8. PMC10162651 37147415

[7] Dahlke S, Butler JI, Toubiana M, Shrestha S, Baskerville K, Devkota R, et al. Understanding older adults’ experiences with cannabis for medicinal purposes: a mixed methods study. Can J Aging. 2025;44(1):10–9. 10.1017/S0714980824000357. 39618345

[8] Reiman A, Welty M, Solomon P. Cannabis as a substitute for opioid-based pain medication: patient self-report. Cannabis Cannabinoid Res. 2017;2(1):160–6. 10.1089/can.2017.0012. PMC5569620 28861516

[9] Public Health Agency of Canada. Opioid- and stimulant-related harms in Canada. Ottawa: PHAC. 2019 [Cited 2025 Aug 25]. Available from: https://health-infobase.canada.ca/substance-related-harms/opioids-stimulants/.

[10] Rønne ST, Rosenbæk F, Pedersen LB, Waldorff FB, Nielsen JB, Riisgaard H, et al. Physicians’ experiences, attitudes, and beliefs towards medical cannabis: a systematic literature review. BMC Fam Pract. 2021;22:212. 10.1186/s12875-021-01559-w. PMC8532330 34674661

[11] Ng JY, Gilotra K, Usman S, et al. Attitudes toward medical cannabis among family physicians practising in Ontario, Canada: a qualitative research study. CMAJ Open. 2021;9(2):E342–8. 10.9778/cmajo.20200187. PMC8084545 33849983

[12] Philpot LM, Ebbert JO, Hurt RT. A survey of the attitudes, beliefs and knowledge about medical cannabis among primary care providers. BMC Fam Pract. 2019;20:17. 10.1186/s12875-019-0906-y. PMC6341534 30669979

[13] Carlini BH, Garrett SB, Carter GT. Medicinal cannabis: a survey among health care providers in Washington State. Am J Hosp Palliat Care. 2017;34(1):85–91. 10.1177/1049909115604669. 26377551

[14] Syed SA, Singh J, Elkholy H, Rojnić Palavra I, Tomicevic M, Petek Eric A, et al. International perspectives on physician knowledge, attitudes, and practices related to medical cannabis. Front Public Health. 2025;13:1463871. 10.3389/fpubh.2025.1463871. PMC11882600 40051509

[15] Ziemianski D, Capler R, Tekanoff R, et al. Cannabis in medicine: a national educational needs assessment among Canadian physicians. BMC Med Educ. 2015;15:52. 10.1186/s12909-015-0335-0. PMC4374299 25888752

[16] Myran DT, Pugliese M, Roberts RL, Solmi M, Perlman CM, Fiedorowicz J, et al. Association between non-medical cannabis legalization and emergency department visits for cannabis-induced psychosis. Mol Psychiatry. 2023;28(10):4251–60. 10.1038/s41380-023-02185-x. 37500826

[17] Myran DT, Roberts R, Pugliese M, et al. Changes in emergency department visits for cannabis hyperemesis syndrome. JAMA Netw Open. 2022;5(9):e2231937. 10.1001/jamanetworkopen.2022.31937. PMC9482056 36112372

[18] Varin M, Champagne A, Venugopal J, et al. Trends in cannabis-related emergency department visits and hospitalizations among children aged 0–11 years in Canada from 2015 to 2021: spotlight on cannabis edibles. BMC Public Health. 2023;23:2067. 10.1186/s12889-023-16987-9. PMC10591397 37872564

[19] Stall NM, Shi S, Malikov K, et al. Edible cannabis legalization and cannabis poisonings in older adults. JAMA Intern Med. 2024;184(7):840–2. 10.1001/jamainternmed.2024.1331. PMC11106709 38767898

[20] Allan GM, Ramji J, Perry D, Ton J, Beahm NP, Crisp N, et al. Simplified guideline for prescribing medical cannabinoids in primary care. Can Fam Physician. 2018;64(2):111–20. PMC5964385 29449241

[21] Cho YI, Johnson TP, Vangeest JB. Enhancing surveys of health care professionals: a meta-analysis of techniques to improve response. Eval Health Prof. 2013;36(3):382–407. 10.1177/0163278713496425. 23975761

[22] Stillman M, Capron M, Mallow M, Ransom T, Gustafson K, Bell A, Graves D. Utilization of medicinal cannabis for pain by individuals with spinal cord injury. Spinal Cord Ser Cases. 2019;5:66. 10.1038/s41394-019-0208-6. PMC6786285 31632724

[23] Kruger DJ, Gerlach J, Kruger JS, Mokbel MA, Clauw DJ, Boehnke KF. Physicians’ attitudes and practices regarding cannabis and recommending medical cannabis use. Cannabis Cannabinoid Res. 2024;9(4):e1048–55. 10.1089/can.2022.0324. PMC11538087 37098170

[24] Kelley K, Clark B, Brown V, Sitzia J. Good practice in the conduct and reporting of survey research. Int J Qual Health Care. 2003;15(3):261–6. 10.1093/intqhc/mzg031. 12803354

[25] Collins AB, Beaudoin FL, Metrik J, Wightman RS. "I still partly think this is bullshit": a qualitative analysis of cannabinoid hyperemesis syndrome perceptions among people with chronic cannabis use and cyclic vomiting. Drug Alcohol Depend. 2023;246:109853. 10.1016/j.drugalcdep.2023.109853 PMC10121940 36996524

