# Supplementary material for: Emergency department providers’ perspectives on patients’ use of cannabis: therapeutic potential, risks, and prescribing experience
Source: BMC Emerg Med. 2026 Aug 7;26:301. doi: 10.1186/s12873-026-01710-9 (PMC13737515; doi:10.1186/s12873-026-01710-9)
Supplement: Supplementary file 1 [file 12873_2026_1710_MOESM1_ESM.pdf]

Supplementary File 1

Cannabis Opinions Survey (Emergency Department Provider Questionnaire)

Survey instrument: Emergency Department Providers' Perspectives on Patients' Use of Cannabis: Therapeutic Potential, Risks, and Prescribing Experience.
BMC Emergency Medicine
Submission ID 975baacf-9fbe-438f-a98a-7831d3d6da86.

For each statement below, please select the response that best reflects your view.

1. Cannabis should be legalized

- ☐ Strongly agree
- ☐ Agree
- ☐ Neither agree nor disagree
- ☐ Disagree
- ☐ Strongly disagree

2. Cannabis smokers are prone to violence

- ☐ Strongly agree
- ☐ Agree
- ☐ Neither agree nor disagree
- ☐ Disagree
- ☐ Strongly disagree

3. Withdrawal from cannabis can be life threatening

- ☐ Strongly agree
- ☐ Agree
- ☐ Neither agree nor disagree
- ☐ Disagree
- ☐ Strongly disagree

4. Cannabis is a gateway drug (leads to other serious drug use)

- ☐ Strongly agree
- ☐ Agree
- ☐ Neither agree nor disagree
- ☐ Disagree
- ☐ Strongly disagree

5. Cannabis use has a high risk of overdose

☐ Strongly agree
☐ Agree
☐ Neither agree nor disagree
☐ Disagree
☐ Strongly disagree

6. Cannabis is a very dangerous drug

☐ Strongly agree
☐ Agree
☐ Neither agree nor disagree
☐ Disagree
☐ Strongly disagree

7. It is possible that cannabis could have medicinal effects

☐ Strongly agree
☐ Agree
☐ Neither agree nor disagree
☐ Disagree
☐ Strongly disagree

8. It is safer to take prescription painkillers than to use cannabis

☐ Strongly agree
☐ Agree
☐ Neither agree nor disagree
☐ Disagree
☐ Strongly disagree

9. How much risk is there to people who use cannabis?

Legal risk: ☐ No risk ☐ Slight risk ☐ Moderate risk ☐ Great risk
Social risk: ☐ No risk ☐ Slight risk ☐ Moderate risk ☐ Great risk
Health risk: ☐ No risk ☐ Slight risk ☐ Moderate risk ☐ Great risk

10. I believe that medical cannabis should be made available to people with diseases or injuries that could potentially benefit from taking it

☐ Strongly agree
☐ Agree
☐ Neither agree nor disagree
☐ Disagree
☐ Strongly disagree

11. Have you prescribed cannabis for medical use by patients?

- ☐ Yes
- ☐ No
- ☐ I am not a prescriber
